# Matrix Metalloproteinases and Tissue Inhibitors of Metalloproteinases in Extracellular Matrix Remodeling during Left Ventricular Diastolic Dysfunction and Heart Failure with Preserved Ejection Fraction: A Systematic Review and Meta-Analysis

**DOI:** 10.3390/ijms21186742

**Published:** 2020-09-14

**Authors:** Merle M. Krebber, Christian G. M. van Dijk, Robin W. M. Vernooij, Maarten M. Brandt, Craig A. Emter, Christoph D. Rau, Joost O. Fledderus, Dirk J. Duncker, Marianne C. Verhaar, Caroline Cheng, Jaap A. Joles

**Affiliations:** 1 Department Nephrology and Hypertension, University Medical Center Utrecht, P.O. Box 8599, 3508 GA Utrecht, The Netherlands; m.m.krebber-2@umcutrecht.nl (M.M.K.); c.g.m.vandijk-4@umcutrecht.nl (C.G.M.v.D.); r.w.m.vernooij-2@umcutrecht.nl (R.W.M.V.); j.o.fledderus@umcutrecht.nl (J.O.F.); m.c.verhaar@umcutrecht.nl (M.C.V.); k.l.cheng-2@umcutrecht.nl (C.C.); 2 Julius Center for Health Sciences and Primary Care, University Medical Center Utrecht, Utrecht University, P.O. Box 85500, 3508 GA Utrecht, The Netherlands; 3 Experimental Cardiology, Department of Cardiology, Thorax center, Erasmus MC, University Medical Center Rotterdam, P.O. Box 2040, 3000 CA Rotterdam, The Netherlands; m.brandt@erasmusmc.nl (M.M.B.); d.duncker@erasmusmc.nl (D.J.D.); 4 Department of Biomedical Sciences, University of Missouri-Columbia, Columbia, MO 65211, USA; emterc@missouri.edu; 5 Computational Medicine Program, University of North Carolina at Chapel Hill, Chapel Hill, NC 27516, USA; christophrau@unc.edu

**Keywords:** heart failure with preserved ejection fraction, left ventricular diastolic dysfunction, extracellular matrix, fibrosis, matrix metalloproteinase, tissue inhibitor of metalloproteinase, animal models, systematic review

## Abstract

Matrix metalloproteinases (MMPs) and tissue inhibitors of metalloproteinases (TIMPs) are pivotal regulators of extracellular matrix (ECM) composition and could, due to their dynamic activity, function as prognostic tools for fibrosis and cardiac function in left ventricular diastolic dysfunction (LVDD) and heart failure with preserved ejection fraction (HFpEF). We conducted a systematic review on experimental animal models of LVDD and HFpEF published in MEDLINE or Embase. Twenty-three studies were included with a total of 36 comparisons that reported established LVDD, quantification of cardiac fibrosis and cardiac MMP or TIMP expression or activity. LVDD/HFpEF models were divided based on underlying pathology: hemodynamic overload (17 comparisons), metabolic alteration (16 comparisons) or ageing (3 comparisons). Meta-analysis showed that echocardiographic parameters were not consistently altered in LVDD/HFpEF with invasive hemodynamic measurements better representing LVDD. Increased myocardial fibrotic area indicated comparable characteristics between hemodynamic and metabolic models. Regarding MMPs and TIMPs; MMP2 and MMP9 activity and protein and TIMP1 protein levels were mainly enhanced in hemodynamic models. In most cases only mRNA was assessed and there were no correlations between cardiac tissue and plasma levels. Female gender, a known risk factor for LVDD and HFpEF, was underrepresented. Novel studies should detail relevant model characteristics and focus on MMP and TIMP protein expression and activity to identify predictive circulating markers in cardiac ECM remodeling.

## 1. Introduction

Left ventricular diastolic dysfunction (LVDD) is an early common alteration in many cardiovascular diseases (CVDs) and highly prevalent in the general population, with reported incidence ranging from 3% to 39% [1,2]. LVDD leads to elevated LV filling pressures which result from increased chamber stiffness, reduced restoring forces and impaired left atrial (LA) function and LV relaxation [3,4,5]. Clinically, LVDD can remain latent or be accompanied by heart failure (HF) symptoms and deteriorate into HF with preserved ejection fraction (HFpEF) [6,7]. In contrast to HF with reduced ejection fraction (HFrEF) where the LV ejection fraction (LVEF) is <40%, subclinical LVDD and HFpEF patients show a LVEF >50% [8]. It is estimated that around 50% of HF patients suffer from HFpEF, with a two-times higher prevalence in women [9,10], indicating sex-based differences in disease etiology [11,12,13]. Evidence from clinical studies supports the concept that HFrEF and HFpEF have a different pathophysiology [14]. LVDD appears to be a chronic systemic syndrome resulting from CVD co-morbidities [15] which include hypertension and chronic kidney disease (CKD) [16,17], diabetes [18], obesity and metabolic syndrome [19,20] and ageing [21].

LVDD and HFpEF are characterized by systemic inflammation, endothelial (microvascular) dysfunction, impaired intracellular cardiomyocyte calcium handling, cardiac hypertrophy and interstitial fibrosis [22,23]. Fibrosis is a fundamental process in cardiac remodeling and central in development and progression of HF [24]. Following injury, resident cardiac fibroblasts and infiltrating immune cells control extracellular matrix (ECM) composition primarily by secretion of matrix metalloproteinase (MMPs) and tissue inhibitors of metalloproteinases (TIMPs), the inhibitors of MMP proteolytic function [25,26]. Both MMPs and TIMPs can directly impact ECM turnover and homeostasis. Alterations in cardiac expression levels of MMPs and TIMPs have been found in patients with different types of heart disease [27], including idiopathic dilated cardiomyopathy [28,29]. While it was initially thought that MMP activity would limit cardiac fibrosis through ECM protein degradation, new insights have shown that MMPs and TIMPs can directly induce ECM deposition and ECM remodeling based on the type of micro-environment [30]. However, causal data on the role of MMPs and TIMPs in initiation and progression of cardiac fibrosis in LVDD/HFpEF cardiac micro-environment is still lacking.

Despite diagnostic advances, therapeutic approaches known to benefit HFrEF patients have not proven as clinically efficacious for LVDD and HFpEF patients. HFpEF management primarily consists of treatment of co-morbidities, blood pressure control and diuretic treatment but overall, there is poor control of symptoms [31,32]. The use of animal models with specific HFpEF-associated co-morbidities may lead to better understanding of cell-cell and cell-ECM interactions that drive dynamic ECM remodeling. MMPs and TIMPs may have additive value to improve clinical specificity and/or predictive value for LVDD/HFpEF. Circulating levels of MMPs and TIMPs have both been used as prognostic tools in clinical studies [33,34,35] and as potential therapeutic targets [36].

In this systematic review, our aim was to report cardiac MMP and TIMP expression or activity in relation to both LVDD/HFpEF and fibrosis in adequately controlled animal models, e.g., established diastolic dysfunction in absence of systolic dysfunction. Besides providing insights into overall ECM dynamics and patterns of fibrosis, this information may be further used to critically assess (a combination of) novel interesting MMPs and TIMPs as prognostic tools in future studies.

## 2. Results

### 2.1. Study Population Selection and Overall Characteristics

**Figure 1:**
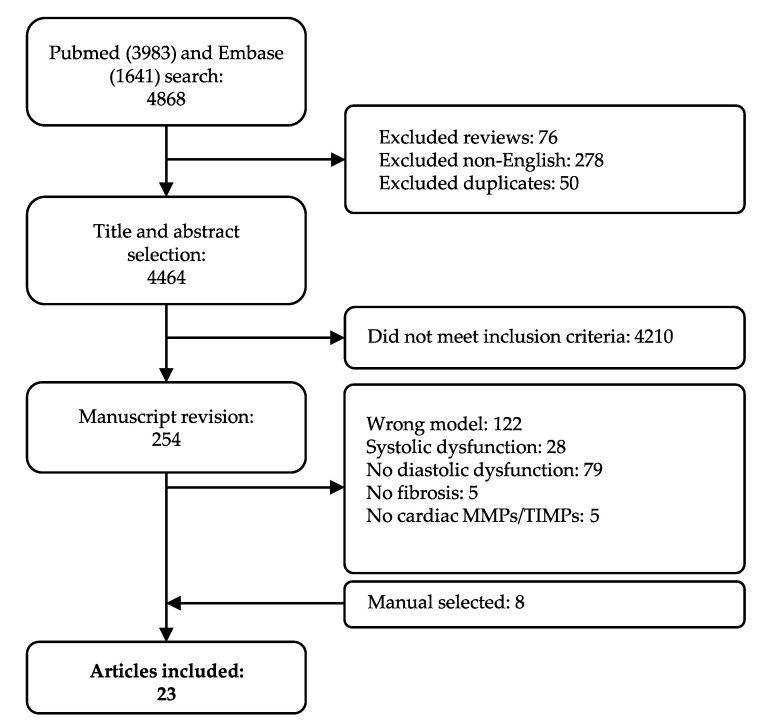
Flow chart of study selection. All articles are included and excluded according to the selection criteria defined in the Materials and Method section.

Our systematic search resulted in 4868 articles. As described in the Materials and Methods Section 4.2, we applied stringent inclusion and exclusion criteria, in order to only cover those phenotypically well-characterized models of HFpEF with established echocardiographic diastolic dysfunction in absence of systolic dysfunction. Studies moreover had to include quantification of fibrosis in cardiac tissue and quantification of at least one cardiac MMP or TIMP. In total, 254 manuscripts were screened on full-text, and 239 articles were excluded per exclusion criteria, of which 28 included systolic dysfunction. Eight articles were added after cross-referencing. Finally, data was extracted from 23 articles (Figure 1). We observed a large variety in overall study characteristics (Supplementary Materials Table S1). The majority of studies used rodents: either mice (12 articles) [37,38,39,40,41,42,43,44,45,46,47,48] or rats (6 articles) [49,50,51,52,53,54]. Other species included swine (n = 3) [55,56,57], rabbit (n = 1) [58] and guinea pig (n = 1) [59]. All but one of the studies employing mice used a C57BL/6 strain or adapted strains with a C57BL/6 background. Rat models showed more heterogeneity; Wistar (Han), spontaneously-hypertensive (SHR), Dahl salt-sensitive (SS) and ZSF1. All three swine models were a different strain. Eighteen articles reported the animal’s sex, while the remainder did not specify the sex or, in one case, used both sexes. Only few of the included articles (5/23) focused on female animals. Various articles studied more than one underlying co-morbidity for LVDD/HFpEF. For example, Brandt et al. studied LVDD in male lean and obese rats with and without deoxycorticosterone acetate (DOCA)-induced hypertension. They showed significant changes in LVDD in three relevant comparisons, e.g., lean vs. obese (metabolic alteration), lean + DOCA vs. obese + DOCA (metabolic alteration) and obese vs. obese + DOCA (hemodynamic alteration) (Table S1) [49].

LVDD was primarily examined using (tissue Doppler) echocardiography E/A ratio (15/23), followed by changes in peak E-wave velocity (8/23), E/e’ ratio (6/23) or isovolumic relaxation time (IVRT, 7/23). Invasive hemodynamic measurements end diastolic pressure (EDP) (11/23), minimum derivative of pressure over time (dP/dt_min_) (7/23), time constant of relaxation Tau (6/23) and end diastolic pressure volume relationship (EDPVR) (5/23) were reported less frequently (Figure S1A). Moreover, two studies included strain measurements using speckle tracking echocardiography. Due to the low number of studies, no meta-analysis was performed. Quantification of fibrosis primarily focused on protein; collagen content using immunohistochemical (IHC) staining, mainly Sirius Red (SR) (12/23) or hydroxyproline assay (Figure S1B,C). MMP tissue activity by gelatinase assay was measured in about half of the articles (11/23) (Figure S2A). These 11 articles assessed MMP2, or variants, while MMP9 was quantified 5 times (Figure S2B). MMP tissue protein levels were primarily quantified by western blot (WB), and focused on MMP9 (4/23) (Figure S2C). Most articles reported mRNA expression of MMP9 (13/23), MMP2 (12/23), or TIMP1 (12/23) (Figure S2D). Extracted data of all studies can be found in Table S2 (cardiac outcomes), Table S3 (fibrotic outcomes), and Table S4 (MMP and TIMP outcome).

### 2.2. Quality Assessment of the Studies

The majority of studies reported the animal details such as strain, sex and age adequately (Figure S3). Of note, only 40% of the studies reported random allocation or stratification of the animals. Baseline characteristics regarding echocardiographic parameters and blinded data processing and analysis were reported infrequently.

### 2.3. Meta-Analysis on Diastolic Function and Fibrosis in Models of LVDD/HFpEF

Concerning diastolic function, all included studies showed similar ejection fraction (EF), fractional shortening (FS) and/or peak derivative of pressure over time (dP/dt_max_) in the experimental model and control, as defined in the exclusion criteria. Studies were first divided based on underlying pathophysiology; ageing (3 comparisons), hemodynamic alterations (17 comparisons) and metabolic alterations (16 comparisons) (Table S1). Due to the low number of comparisons, i.e., three, all in mice, no meta-analysis was performed for ageing. All relevant directional changes, standard mean differences (SMDs) and confidence intervals (CIs) resulting from meta-analysis are available in Table 1 and Table S5 respectively.

**Figure 2:**
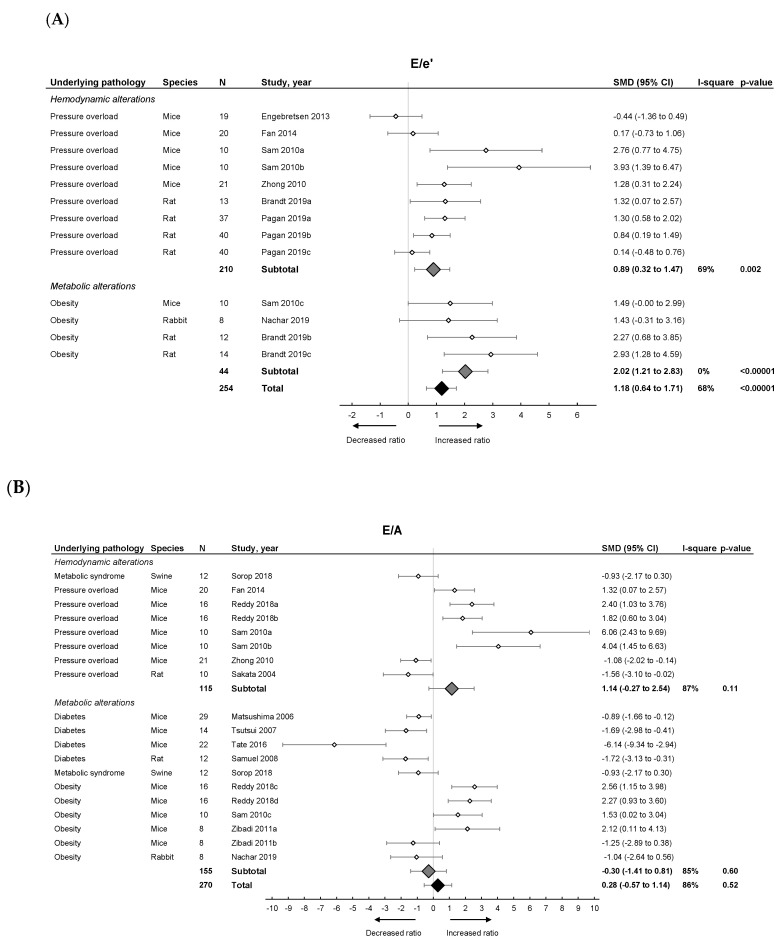
The effect of LVDD/HFpEF on cardiac parameters E/e’ (panel (**A**)) and E/A (panel (**B**)). Forrest plot; the right side shows an increased ratio in LVDD/HFpEF animals, the left side shows a decreased ratio in LVDD/HFpEF animals. Data are presented as standard mean differences (SMDs) with 95% CI. Arrows indicate increased and decreased E/e’ ratio (**A**), and increased and decreased E/A ratio (**B**) respectively. Only the first author of each study is shown; multiple comparisons within one study are shown with a, b, c or d and correspond with the study overview (Table S1). CI, confidence interval; E/A, ratio between peak early diastolic transmitral velocity (E) and late (atrial) transmitral flow velocity (A); E/e’, ratio between peak early diastolic transmitral velocity (E) and early diastolic mitral annular velocity (e’); I^2^, measurement of heterogeneity; N, cumulative sample size; SMD, standardized mean difference.

Pooled analysis of E/e’ (Figure 2A) but not E/A (Figure 2B) ratios showed an overall increase in LVDD/HFpEF. There was no pooled effect on E-wave or IVRT. E/e’ alone moreover significantly increased in both models with metabolic alterations having a higher E/e’ ratio (subgroup difference *p* = 0.03). For E/A, E-wave and IVRT, there were no subgroup differences.

**Figure 3:**
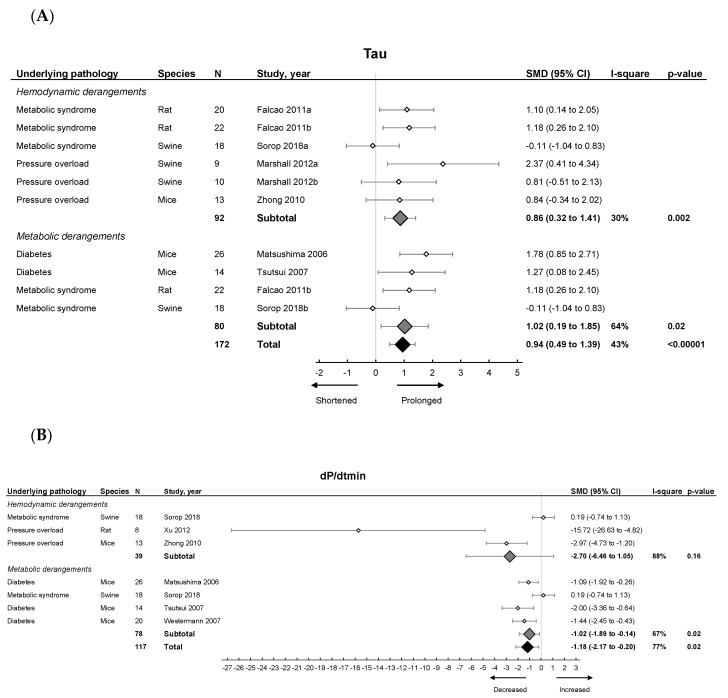
The effect of LVDD/HFpEF on cardiac parameters Tau (panel (**A**)) and dP/dt_min_ (panel (**B**)). Forrest plot; the right side shows an increased effect in LVDD/HFpEF animals, the left side shows a decreased effect in LVDD/HFpEF animals. Data are presented as SMDs with 95% CI. Arrows indicate shortened and prolonged time constant of relaxation Tau (**A**), and decreased and increased rate of pressure change dP/dt_min_ (**B**) respectively. Only the first author of each study is shown; multiple comparisons within one study are shown with a, b, c or d and correspond with the study overview (Table S1). CI, confidence interval; I^2^, measurement of heterogeneity; N, cumulative sample size; SMD, standardized mean difference; dP/dt_min_, minimum rate of pressure change; Tau, time constant of ventricular relaxation.

Invasive hemodynamic measurements represented by EDP showed an increased pressure in HFpEF, EDPVR increased in slope and Tau showed a prolonged relaxation duration (Figure 3A). dP/dt_min_ (Figure 3B) showed overall reduced maximal rate of fall of LV pressure. Subgroup analysis revealed that EDPVR and Tau increased in both hemodynamic and metabolic models, without subgroup differences (*p* = 0.89 and *p* = 0.76). EDP remained unchanged in subgroup analysis and was similar in both models. dP/dt_min_ decreased in metabolic models, without subgroup differences (*p* = 0.39). Thus hemodynamic and metabolic models generally display similar changes in cardiodynamics (Table 1).

**Figure 4:**
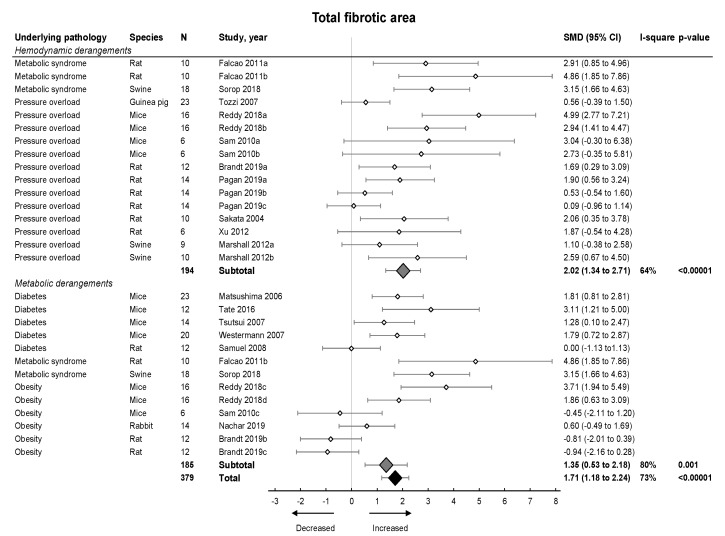
The effect of LVDD/HFpEF on total fibrotic area. Forrest plot; the right side shows an increased effect in LVDD/HFpEF animals, the left side shows a decreased effect in LVDD/HFpEF animals. Data are presented as SMDs with 95% CI. Arrows indicate increased and decreased fibrotic percentage area respectively. Only the first author of each study is shown; multiple comparisons within one study are shown with a, b, c or d and correspond with the study overview (Table S1). CI, confidence interval; I^2^, measurement of heterogeneity; N, cumulative sample size; SMD, standardized mean difference.

**Figure 5:**
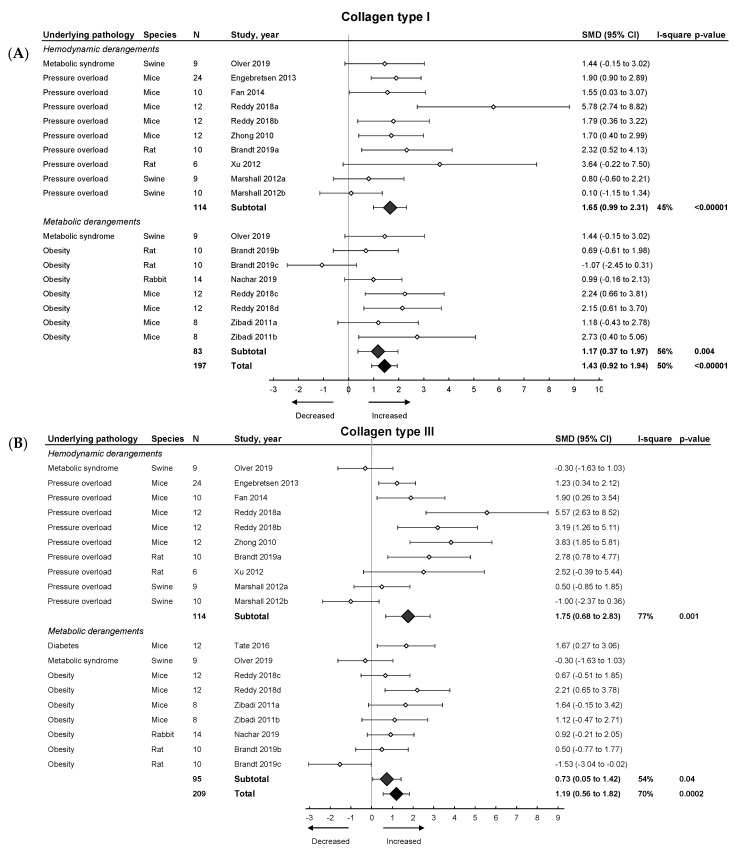
The effect of LVDD/HFpEF on collagen type 1 (panel (**A**)) and collagen type 3 (panel (**B**)) mRNA levels. Forrest plot; the right side shows an increased effect in LVDD/HFpEF animals, the left side shows a decreased effect in LVDD/HFpEF animals. Data are presented as SMDs with 95% CI. Arrows indicate increased and decreased Collagen type 1 (**A**), and increased and decreased type 3 (**B**) mRNA expression respectively. Only the first author of each study is shown; multiple comparisons within one study are shown with a, b, c or d and correspond with the study overview (Table S1). CI, confidence interval; COL1, collagen type 1; COL3, collagen type 3; I^2^, measurement of heterogeneity; N, cumulative sample size; SMD, standardized mean difference.

Subsequently, we focused on fibrosis. An overview of meta-analyses outcomes for fibrosis can be found in Table 1 and Table S6. Note the relative paucity of data on collagen protein as compared to mRNA levels (Table S6). Meta-analysis on positive percentage area as assessed by IHC showed a pooled increase (Figure 4). Both hemodynamic and metabolic models were associated with an increase, without subgroup differences (*p* = 0.22), resulting from increased collagen type I expression (mRNA and protein) and increased collagen type III on mRNA but not protein level (Figure 5A,B).

#### 2.3.1. Meta-Analysis on MMPs and TIMPs in Pooled Models of LVDD/HFpEF

**Figure 6:**
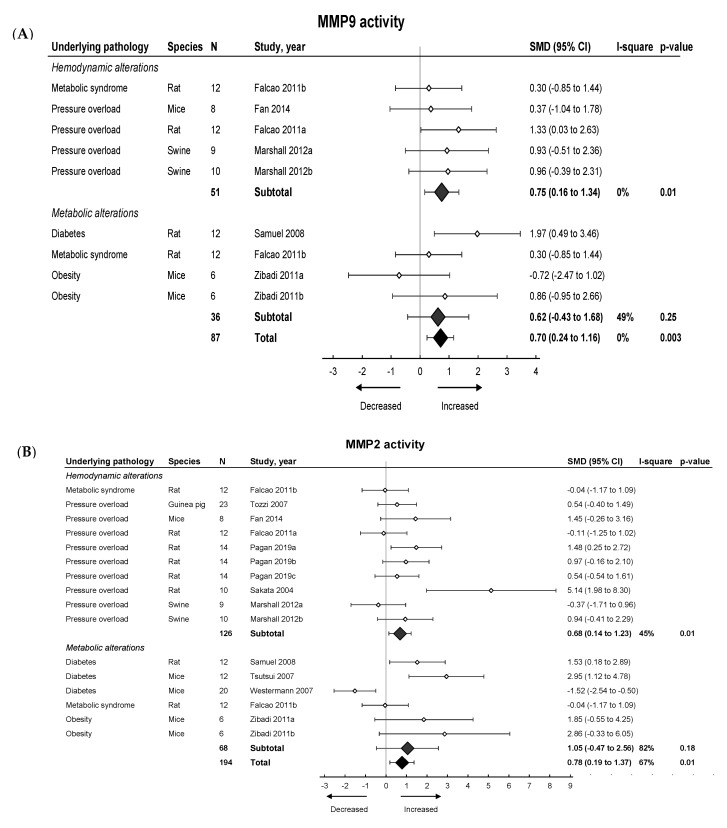
The effect of LVDD/HFpEF on MMP2 (panel (**A**)) and MMP9 (panel (**B**)) activity. Forrest plot; the right side shows an increased effect in LVDD/HFpEF animals, the left side shows a decreased effect in LVDD/HFpEF animals. Data are presented as SMDs with 95% CI. Arrows indicate increased and decreased MMP2 (**A**), and increased and decreased MMP9 (**B**) enzyme activity respectively. Only the first author of each study is shown; multiple comparisons within one study are shown with a, b, c or d and correspond with the study overview (Table S1). CI, confidence interval; I^2^, measurement of heterogeneity; MMP, matrix metalloproteinase; N, cumulative sample size; SMD, standardized mean difference; TIMP, tissue inhibitor of metalloproteinase.

We then investigated the pooled effects of LVDD/HFpEF on MMP and TIMP expression and activity. An overview of meta-analyses outcomes for all MMPs and TIMPs can be found in Table 1 and Table S7. Note the paucity of data on MMP and TIMP protein as well as MMP zymography as compared to mRNA levels (Table S7). For mRNA expression, MMP2, -8, -9, -11, -12, -14, -15 and TIMP1, -2, -3, -4 were investigated. We found no pooled changes in MMP or TIMP expression in LVDD/HFpEF, except for decreased MMP15 and increased TIMP1 expression. Protein levels of MMP2, -9, TIMP1 and -2 were subsequently analyzed. Pooled MMP2, TIMP1 and TIMP2 protein expressions were similar but MMP9 increased. LVDD/HFpEF increased zymographic activity of MMP2 and MMP9 (Figure 6A,B).

#### 2.3.2. Meta-Analysis on MMPs and TIMPs in Models Involving Hemodynamic and Metabolic Alterations

Hemodynamic models showed no changes in MMP2, -8 -9, -14, -15, TIMP2, -3 and -4 mRNA and TIMP1 protein expression, but MMP2 and TIMP1 protein expression increased. MMP2 and MMP9 protein expression also increased but were only measured in one study [42]. TIMP2 protein and MMP2 and MMP9 zymographic activity increased (Figure 6, Table 1 and Table S7). Metabolic models showed no changes in MMP2, -8, -9, -11, -14, -15, TIMP1, -2 and -3 mRNA expression. There was a decrease in TIMP4 mRNA. MMP2 protein was only measured in 1 study and decreased while TIMP1 and TIMP2 protein remained unchanged [42]. MMP9 protein levels increased. Both MMP2 and MMP9 zymographic activity were similar in metabolic models versus controls (Figure 6, Table 1 and Table S7)

#### 2.3.3. Descriptive Effect on Models Involving Ageing (All in Mice)

Chiao et al. [37] but not Ma et al. [38] showed increased fibrotic percentage area. However, both studies reported decreased collagen I and/or collagen III mRNA. Thus, cardiac fibrosis in ageing, at least in mice, in contrast to the induced hemodynamic and metabolic models, was not due to increased collagen synthesis. Ageing was associated with decreased MMP8 and MMP9 [37] and MMP28 protein [38]. There were no changes in other MMPs or TIMPs.

## 3. Discussion

Due to the high morbidity and mortality associated with HFpEF [60] there is an urgent need for additive and predictive circulating markers and early detection of changes in structural and functional cardiac parameters. In our systematic review concerning animal models of LVDD/HFpEF and cardiac fibrosis in relation to MMPs and TIMPs, we aimed to identify patterns associating ECM dynamics with LVDD and HFpEF pathology. We included 23 studies with a large range of study characteristics and our assessment indicated relatively low quality with respect to random allocation and blinded assessment of results. The relative heterogeneity of study characteristics partially reflects clinical findings since HFpEF is a multifactorial disease and an overarching pathology resulting from a variety of underlying CVD co-morbidities [31]. Overall, there was a sex-based bias towards male gender and bias towards pressure overload and metabolic models of LVDD/HFpEF. Our main findings show that echocardiographic measurements of LVDD/HFpEF, including E/A, E-wave and IVRT, do not consistently relate with accepted phenotypic criteria of the current established experimental models of LVDD/HFpEF. Invasive hemodynamic measurements such as Tau, EDP, EDPVR and dP/dt_min_, on the other hand, seem to associate more closely with the phenotype. Regarding cardiac expression of MMPs and TIMPs, it appears highly unlikely that the presence or activity of a single MMP or TIMP may hold the key to diagnosing or even treating a multifactorial disease such as HFpEF. We identified MMP15 and increased TIMP1 mRNA and MMP9 protein expression in LVDD/HFpEF. Increased MMP2 and MMP9 zymographic activity both associated with pooled LVDD/HFpEF.

### 3.1. Echocardiography and Tissue Doppler Parameters of LVDD and HFpEF

For our study inclusion, we selected and prioritized cardiac parameters in accordance with the current American Society of Echocardiography and the European Association of Cardiovascular Imaging (ASE/EACVI) guidelines [4]. While LV cardiac pressure catheterization is the gold standard for evaluating EDPVR, dP/dt_min_ and Tau, in the clinic both LVDD and HFpEF are primarily diagnosed using echocardiography [3,61,62]. Assessing LA strain by speckle tracking echocardiography has recently also emerged as a relevant non-invasive clinical alternative, circumventing the time-consuming measurements associated with tissue Doppler [62,63,64]. In clinical practice, measurements in patients with normal EF currently include e’ and E/e’ ratio to estimate LV filling pressure. The interpretation of the E-wave, A-wave and e’ however depend on strictly defined thresholds; decreased E/A ratio (<0.8) reflects the compensatory increase in late atrial filling when the LV fails to relax, primarily linked to alterations in early LVDD [3,5]. To the best of our knowledge, such thresholds have not been clearly set for experimental animals. In the current study, E/A ratios were still the most frequently used to assess diastolic function. However, our meta-analysis on pooled effects showed that this ratio is not consistently altered. This may be partially explained by the fact that this ratio is highly afterload-dependent [65,66] and the majority of our models involved a hypertensive background (20/36 comparisons). Previously it was found that E/A ratios in murine models of HFpEF were difficult to measure due to high heart rates [67]. In general, anesthetic agents influence diastolic function in healthy mice [68]. Among others, inhaled anesthetics reduce afterload beneficially [69] but changes may be less evident in HF models [70]. Almost half of the included studies (11/23) performed echocardiography or tissue Doppler under isoflurane (analogues) and it remains pivotal for obtaining accurate measurements. The general impact of anesthetics on perioperative LVDD and HFpEF remains unclear [71].

Given the pooled and separate effect of hemodynamic models on increased E/e’ ratio but lack of effect on E/A and E-wave, e’ seems to represent the most reliable change in LVDD/HFpEF. Indeed, Zhong et al. [43], Pagan et al. [51] and Sam et al. [42] show a decreased e’ (3/5 comparisons). Clinically, e’ also has the highest reproducibility and a consistent association with CVD outcomes [62].

Invasive hemodynamics were less frequently applied in the included articles, probably due to practical constraints, especially in small animals. In pooled data, we did find prolonged Tau and decreased dP/dt_min_, which were both identified in metabolic alterations, in accordance with literature [72,73].

### 3.2. Influence of Fibrosis on Development and Progression of LVDD and HFpEF

The cardiac ECM mainly comprises fibrillar collagen, specifically collagen type I and III (85–90% to 5–11%, respectively) [25]. Myocardial stiffness in patients with HFpEF is associated with increased collagen type I expression and cross-linking [74]. Besides cardiac (myo) fibroblasts, other cardiac cell types contribute to excess ECM accumulation by either ECM secretion [75] or differentiation to myofibroblasts [76,77]. Animal models have shown that cardiac fibroblasts are activated early in development of LVDD, leading to collagen deposition and activation of the cardiac renin-angiotensin-aldosterone system (RAAS), driving inflammatory processes and TGF-β signaling [78]. Our meta-analysis showed that HFpEF is associated with an overall increase in positive fibrotic area. Both hemodynamic and metabolic alterations associated with increased fibrotic area. Transcriptome analysis on lateral LV wall biopsies of HFpEF patients indeed showed upregulation of collagen 1α1 and collagen 3α1, among others [79].

### 3.3. MMP and TIMP Activity in LVDD and HFpEF

Several clinical studies have previously tried to improve LVDD and HFpEF diagnosis by incorporating plasma markers of collagen turnover [80,81,82]. The majority of our included studies investigating the relation between HFpEF and MMPs focused on MMP2, MMP9 and TIMP1 mRNA expression. Our overall meta-analysis showed increased MMP2 and MMP9 activity, MMP9 protein, TIMP1 gene expression and decreased MMP15 gene expression. RNA-sequencing of atrium of high-salt fed rats however showed increased MMP15 levels [83] emphasizing the need to further study this MMP in both hemodynamic and metabolic models of HFpEF. In general, increases in plasma levels of MMP2, MMP9 [80] and TIMP1 [84] have been found in HFpEF patients with a hypertensive background. A transcriptomic study on lateral LV wall biopsies of HFpEF previously showed a decreased MMP15 gene expression [79]. MMP gene expression may be determined by different external factors and may be cell type and ECM-specific [85,86]. Moreover, both MMPs and TIMPs are heavily regulated at mRNA, protein and activity levels. Interpreting MMP and TIMP activity in LVDD/HFpEF solely based on mRNA levels therefore is not directly translatable to clinical settings. While previous studies have confirmed that a ratio of 1:1 exists for the breakdown product of collagen type I, procollagen type I C-terminal propeptide (PICP), in the bloodstream versus (cardiac) collagen type I production, this seems to be less established for cardiac MMP and TIMP activity versus their circulating levels. Zhang et al. employed a rodent model of aortic stenosis-induced pressure overload and while they did not report MMP or TIMP cardiac tissue mRNA or protein levels, 8 weeks after induction of pressure overload, MMP1, MMP2, MMP9 and TIMP1 protein levels were significantly increased in the circulation compared to time-matched controls [87]. In streptozotocin (STZ)-induced diabetic minipigs, both pro- and active MMP2 and MMP9 zymography in the LV decreased compared to control animals [88]. This finding was in accordance with decreased serum protein levels of MMP2 and MMP9. Protein levels as measured by WB and IHC of these MMPs, however, showed no changes in expression while mRNA levels for MMP9 even increased in diabetic animals [88]. These data also indicate dissimilarities between mRNA and protein expression and MMP tissue enzymatic activity. Changes in active MMPs seem to most closely resemble serum values.

Differences in MMP and TIMP expression and activity may also be relevant in relation to underlying co-morbidities and severity of HFpEF. While our meta-analysis had a low power concerning subgroup analysis, we did identify higher MMP2 and MMP9 protein in hemodynamic and lower TIMP4 gene expression in metabolic alterations. Sakamuri et al. previously studied high-fat diet changes in TIMP4 knock-out (KO) mice compared to wild-type. TIMP4 KO mice showed reduced cardiac fibrosis and systemic protection from dyslipidemia, indicating a protective mechanism in the context of metabolic changes [89]. In chronic HF settings, epigenetic changes could be a relevant mode of action. In a mouse model of aorta-vena cava fistula, methylation of the TIMP4 promotor was shown. TIMP4 directly regulates MMP9 and indeed MMP9 protein was upregulated in the mouse model [90], in accordance with our findings; MMP9 protein showed significant upregulation in hemodynamic compared to metabolic models. No conclusions on MMP2 protein in metabolic alterations could be drawn, since they were only assessed in one study [42]. Similar results were found by Ahmed et al., where MMP-9 levels were elevated in hypertensive patients with LVH and HFpEF and hypertensive LVH patients but not in hypertensive controls [91]. Contrarily, MMP2 levels decreased in hypertensive LVH patients without HFpEF [91]. Assessing circulating MMP and TIMP levels in relation to HFpEF could aid physicians in determining whether a certain co-morbidity primarily drives disease progression in a particular patient. Note that the chosen end-point of experimental studies will certainly influence fibrotic progression. Thus, even within the pathology of LVDD and HFpEF, severity may directly relate to MMP and TIMP dynamics and ECM turnover.

### 3.4. Study Limitations

We retrieved 23 relevant studies via our systematic search, complemented by cross-referencing. In order to exclusively include models with well-established phenotypic characterization, we applied stringent inclusion criteria. These included established echocardiographic measurements of diastolic function in absence of systolic dysfunction, combined with quantification of fibrosis and cardiac tissue quantification of at least one MMP or TIMP, and only in pre-determined experimental models known to represent co-morbidities in human HFpEF. Inclusion of stable LVDD/HFpEF models came at the cost of the relatively low power of our meta-analysis. Our broad search strategy was performed in two biomedical databases, leading to a large number of references. Several papers did not explicitly mention either LVDD/HFpEF or MMP/TIMP expression while focusing on disease development or only retrieved MMPs/TIMPs by applying an mRNA-sequencing protocol. Consequently, these studies could not be identified by our search, but we have resolved this by cross-reference searching.

Several studies including relevant co-morbidities were excluded based on a decrease in systolic function. While a threshold to discern HFrEF from HFpEF is routine in clinical practice [4], this does not automatically hold true for experimental models. We therefore excluded all studies (28/239) showing significant differences in systolic function, e.g., EF, FS or dP/dt_max_, compared to controls. On the other hand, clinical diagnosis of LVDD or HFpEF is described in detailed guidelines and depends on specific alterations in cardiac parameters that are not well-defined in animal models. We therefore included all studies that showed a significant difference in at least one measured clinically relevant diastolic parameter, e.g., E/e’, E/A, Tau and dP/dt_min_, compared to control, irrespective of the direction of the change. We also identified significant heterogeneity (>75%) between several comparisons. This can be largely explained by differences in study design, cardiac, fibrotic and MMP and TIMP outcome as well as the differences between underlying pathology, animal species and strains. Creating a division between hemodynamic and metabolic-driven pathologies allowed us to analyze both overall data and individual underlying pathologies, in line with the heterogeneity of co-morbidities found in HFpEF patients [92,93]. By including more than one comparison for several studies, controls may be over-analyzed which could affect the pooled outcome but to lesser extent the subgroups. Moreover, most studies did not specify which part of the myocardium was used for fibrotic or MMP/TIMP analysis, probably accounting for some of the differences in outcome.

## 4. Materials and Methods

We registered the systematic review protocol in PROSPERO (CRD4202018315) on 27 May 2020.

### 4.1. Literature Search

A systematic search MEDLINE and Embase was conducted from database inception up to March 2020. Medical Subject Headings (MeSH) terms and free text terms in title and abstract were used to identify all possible studies regarding HFpEF and LVDD with measured (diastolic) heart function, fibrosis and MMP or TIMP measurements. The search syntax can be found in Table S8.

### 4.2. Study Selection

Titles and abstracts were evaluated independently by two researchers (C.G.M.v.D and M.M.K.). Duplicates, non-English, editorials, poster presentations, letters or abstracts only were excluded prior to full text assessment. Consequently, all articles deemed eligible in the title and abstract screening phase were reviewed in the full-text screening phase, independently and in duplicate. The two reviewers resolved disagreements by discussion and, if needed, by third-party adjudication. Only animal studies focusing on stable HFpEF or LVDD and not progressive models leading to HFrEF were included. Inclusion and exclusion criteria for all different animal models and cardiac parameters were predefined and listed below:

Pathologies eligible for inclusion: (1) amyloid (non-hereditary) cardiomyopathy, (2) hypertrophic cardiomyopathy independent of coronary artery disease (CAD) and myocardial infarction (MI), (3) all models of trans-aortic constriction (TAC) in absence of effects on ejection fraction (EF) and fractional shortening (FS), e.g., 2-kidney-1-clip (2K1C) and abdominal-aortic banding, (4) aortic stenosis in absence of CAD or MI, (5) atrial fibrillation in exercise in absence of CAD or MI, (6) pulmonary hypertension, (7) chronic (e.g., osmotic pump-induced) angiotensin II (AngII), (8) chronic (e.g., osmotic pump-induced) deoxycorticosterone acetate (DOCA)-salt, (9) chronically induced isoproterenol, (10) mitral (non-hereditary) regurgitation, (11) arterio-venous fistula (AVF), (12) natural ageing and (13) (genetic) models not restricted to rodents described by Valero-Muñoz et al. [11].

Pathologies that were excluded: (1) stenotic or hypertensive models where the underlying cause is based on systemic atherosclerosis and/or atherosclerotic coronary artery disease (CAD) since CAD is seen as a macrovascular disease and onset mechanisms may deviate from true LVDD and HFpEF, (2) unstable HFpEF of LVDD models that eventually progress into HFrEF (e.g., early phase MI), (3) trained ischemia models such as ischemia-reperfusion (I/R), (4) genetic models of dilated cardiomyopathy (DCM), (5) Homocysteine-enriched diets, (6) exclusion criteria in accordance with HELPFUL protocol [6]. (7) LVDD in combination with HFrEF was also excluded [11] as well as (8) animals with localized genetic alterations prior to introduction of diastolic heart failure.

Studies that met the criteria were further assessed and only included if; (1) HFpEF or LVDD was confirmed with at least one parameter of diastolic function in accordance with the ASE/EACVI guidelines [4], (2) fibrosis was confirmed at mRNA or protein (e.g., Western Blot or immunohistochemistry) level, and (3) MMP and/or TIMP activity was confirmed at protein level, preferentially using gelatin zymography. Changes in levels of fibrosis and MMP/TIMP (over time) measured using mRNA were also included. After this final round, all articles that met the criteria were well cross-referenced to ascertain that all relevant articles were included.

### 4.3. Quality Assesment

Methodological quality assessment of the included studies was performed by a risk of bias tool adapted from Papazova et al. [94]. We separated animal characteristics in specified questions addressing each detail. Furthermore, we divided the blinded assessor for the histological (fibrosis) outcome and echocardiography. Studies were labeled as positive (yes), negative (either partially addressed or not mentioned (N.M.)) or not applicable (N.A.).

### 4.4. Data Extraction

Using standardized piloted data-extraction forms, pair of reviewers independently extracted data on study characteristics including species, strain, sex, age, weight, number of animals and experimental model. The total duration of the experiment was reported as end time point. Cardiac parameters from either echocardiography, invasive hemodynamics or tissue Doppler were extracted for (1) diastolic function and, when applicable, (2) systolic function. Fibrotic outcomes and MMP and/or TIMP outcomes were extracted from all parameters measured. Studies that only showed representative images of a staining or WB related to fibrosis or collagen or zymography but no quantitative data were excluded. For each outcome, the sample size and standard deviation (SD) or standard error (SEM) were extracted. When the sample size was described as a range, the lowest number of replicates was used. When data was not present in text or tables, graphical data was extracted using WebPlotDigitizer (https://automeris.io/WebPlotDigitizer/) by one researcher (C.G.M.v.D) and validated using PlotDigitizer (http://plotdigitizer.sourceforge.net/) by a second researcher (M.M.K.).

### 4.5. Data Analysis

SEM of all extracted data was transformed to SD. Extracted data of cardiac outcome, fibrotic outcome and MMP/TIMP outcome were converted to their effect size and displayed as standardized mean differences (SMD), defined as the between-group difference in mean values divided by the pooled SD, with their corresponding 95% confidence interval using Review Manager (version 5.3.5). Studies were divided based on underlying pathophysiology; ageing, hemodynamic alterations and metabolic alterations.

We examined the heterogeneity by visually inspecting forest plots for the presence of heterogeneity and the tau^2^ and I^2^ statistics as a measure of between-study heterogeneity. The I^2^ described a percentage of variation across the studies attributable to heterogeneity with values of <25%, 25–75%, and >75% interpreted as, respectively, low, moderate, and high between-trial heterogeneity. We used standard inverse-variance random-effect meta-analysis to combine outcome data across studies on predetermined parameters [95] in Review Manager (Version 5.3.5).

This systematic review followed the PRISMA guidelines (Table S9).

## 5. Conclusions

Our study shows that when MMPs and TIMPs are studied in relation to LVDD/HFpEF, cardiac mRNA expression is still most frequently measured while this does not seem to resemble cardiac ECM dynamics in these experimental models. Since post-transcriptional and post-translational activation of both MMPs and TIMPs takes place, future studies should focus on MMP and TIMP protein levels and enzyme activity. Changes in active MMPs seem to most closely resemble serum values. Besides increased enzymatic activity of MMP2 and MMP9 and TIMP1 mRNA, we propose MMP15 as an interesting novel candidate in HFpEF-driven cardiac fibrosis, as MMP15 mRNA was downregulated in HFpEF compared to controls. Ideally, a combination of tissue and plasma concentration should be measured to correlate MMP and TIMP dynamics for a better clinical translatability. Furthermore, MMP and TIMP protein expression and enzymatic activity may differ in underlying co-morbidities associated with LVDD/HFpEF; we identified TIMP4 mRNA as a relevant candidate since it was downregulated in metabolic compared to hemodynamic models.

Besides these conclusions related to MMPs and TIMPs, a number of general recommendations related to experimental LVDD/HFpEF studies can be put forward. These are listed below.

## 6. Recommendations for Future Studies on LVDD/HFpEF

We recommend future studies to focus on experimental LVDD/HFpEF models in which female gender is separately represented, on models that include pure volume overload and atrial fibrillation and on models of ageing and ageing in combination with either hemodynamic or metabolic models. Perform adequate hemodynamic and metabolic phenotyping to more clearly discern differences between LVDD/HFpEF associated sub-groups. Focus should be on measuring invasive hemodynamic parameters instead of, or in addition to, (speckle tracking) echocardiography, since these appear to be more reliable across species and will decrease the translation bias to the clinic. Include a systolic parameter, in addition to establishing diastolic dysfunction, to ascertain pure LVDD/HFpEF. Lastly, we recommend a focus on spatiotemporal patterns of diastolic dysfunction and fibrosis, to ascertain whether clinical stages of LVDD/HFpEF are translatable to experimental models.

## Figures and Tables

**Table 1:**
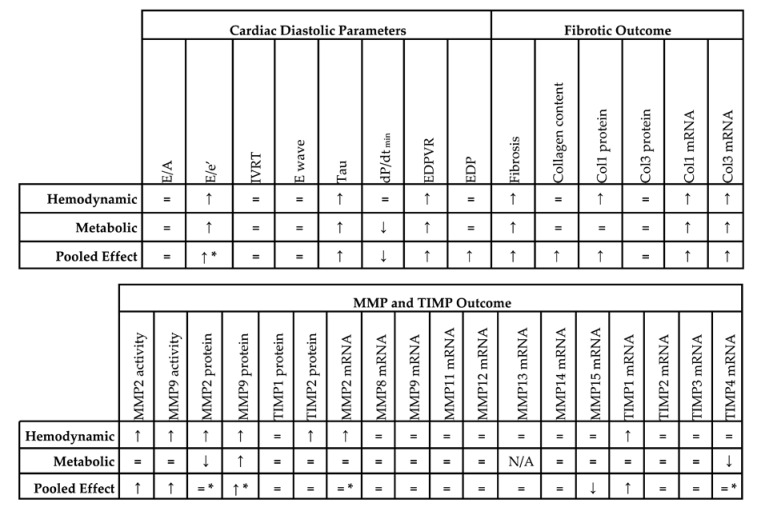
Summarizing table of meta-analysis.

↑, effect is higher in LVDD/HFpEF; ↓, effect is lower in LVDD/HFpEF; =, no significant effect; *, significant subgroup difference. Col, collagen; dP/dt_min_, minimum rate of pressure change; E/A, ratio between peak early diastolic transmitral velocity (E) and late (atrial) transmitral flow velocity (A); E wave, peak early diastolic transmitral velocity; E/e’, ratio between peak early diastolic transmitral velocity (E) and early diastolic mitral annular velocity (e’); EDP, end diastolic pressure; EDPVR, end diastolic pressure volume relationship; IVRT, isovolumetric relaxation time; MMP, matrix metalloproteinase; N/A, not available; Tau, time constant of ventricular relaxation; TIMP, tissue inhibitor of metalloproteinase.

## Supplementary Materials

The following are available online at https://www.mdpi.com/1422-0067/21/18/6742/s1. Figure S1 shows a graphical representation of cardiac and fibrotic outcomes. Figure S2 shows a graphical representation of MMP and TIMP outcomes. Figure S3 shows the quality assessment score of all included articles. Table S1 shows the study overview and animal model characteristics of all included studies. Table S2 shows all extracted cardiac outcomes with the interesting diastolic outcomes highlighted. Table S3 shows extracted fibrotic outcomes. Table S4 shows extracted MMP and TIMP outcomes. Table S5 shows a summary of the meta-analysis performed on cardiac outcomes. Table S6 shows a summary of the meta-analysis performed on fibrotic outcomes. Table S7 shows a summary of the meta-analysis performed on MMP and TIMP outcomes. Table S8 shows the exact search string in both MEDLINE and Embase databases. Table S9 shows the PRISMA checklist.

## Abbreviations

AngII	Angiotensin 2
AVF	Arterio-venous fistula
CAD	Coronary artery disease
CI	Confidence interval
CKD	Chronic kidney disease
CVD	Cardiovascular disease
DCM	Dilated cardiomyopathy
DOCA	Deoxycorticosterone acetate
ECM	Extracellular matrix
EDP	End diastolic pressure
EDPVR	End diastolic pressure volume relationship
EF	Ejection fraction
FS	Fractional shortening
HF	Heart failure
HFpEF	Heart failure with preserved ejection fraction
HFrEF	Heart failure with reduced ejection fraction
IHC	Immunohistochemistry
IVRT	Isovolumic relaxation time
LV	Left ventricle
LVDD	Left ventricular diastolic dysfunction
MI	Myocardial infarction
MMP	Matrix metalloproteinase
SHR	Spontaneously-hypertensive rat
SMD	Standardized mean difference
SR	Sirius red
STZ	Streptozotocin
TAC	Transaortic constriction
TIMP	Tissue inhibitor of metalloproteinase
WB	Western blot
