# Supplementary material for: Matrix Metalloproteinases and Tissue Inhibitors of Metalloproteinases in Extracellular Matrix Remodeling during Left Ventricular Diastolic Dysfunction and Heart Failure with Preserved Ejection Fraction: A Systematic Review and Meta-Analysis
Source: Int J Mol Sci. 2020 Sep 14;21(18):6742. doi: 10.3390/ijms21186742 (PMC7555240; doi:10.3390/ijms21186742)
Supplement: Supplementary file 1 [file ijms-21-06742-s001.zip › IJMS Supplemental Material file.docx]

**Supplementary Materials**

A

B

C

**Supplemental Figure S1**. Study characteristics of cardiac and fibrotic outcomes. A) Diastolic parameters measured in number of studies. B) Total number of studies measuring fibrotic outcomes, subdivided in protein analysis (histological staining, WB and collagen content assays) and mRNA analysis (qPCR analysis). C) Different protein targets analysed for fibrotic outcomes. COL1, Collagen type 1; COL3, Collagen type 3; dP/dt_min_, minimum rate of pressure change; E wave, peak early diastolic transmitral velocity; E/A, ratio between peak early diastolic transmitral velocity (E) and late (atrial) transmitral flow velocity (A); E/e’, ratio between peak early diastolic transmitral velocity (E) and early diastolic mitral annular velocity (e’); EDP, end diastolic pressure; EDPVR, end diastolic pressure volume relationship; IVRT, isovolumetric relaxation time; Tau, time constant of ventricular relaxation; WB, western blot.


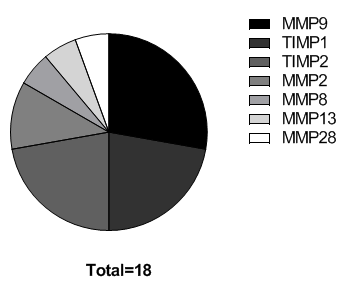

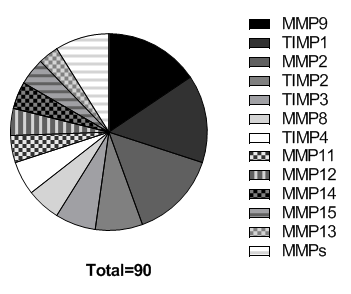

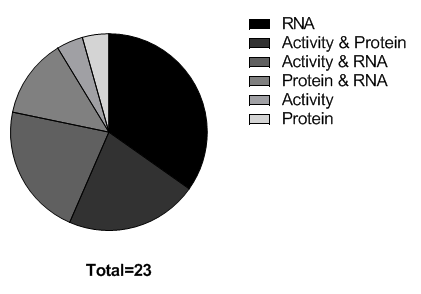

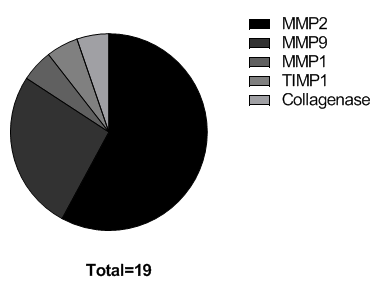

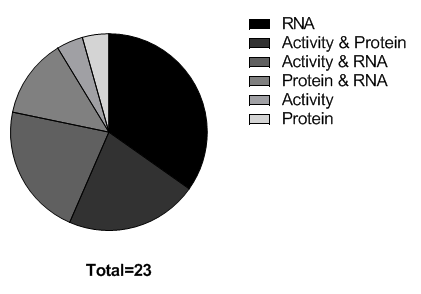

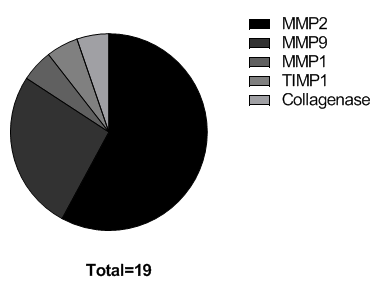

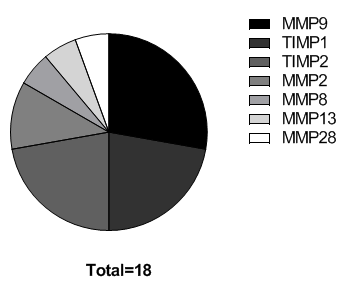


A

B

D


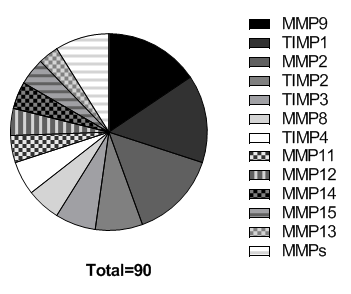


C


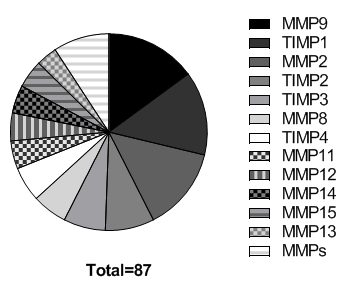


**Supplemental Figure S2.** Study characteristics of MMPs and TIMPs. A) Total number of studies measuring MMP and TIMP outcomes, subdivided in MMP activity, protein analysis (WB, ELISA), mRNA expression (qPCR, mRNA sequencing) or a combination of measurements B) Different MMPs and TIMP1 targets analysed for their enzymatic activity. C) Different MMP and TIMP targets analysed for their protein expression. D) Different MMPs and TIMPs targets analysed for their mRNA expression. Multiple MMP family members were clustered in the part named ‘MMPs’. These MMP family members were not measured often and can be found back in Supplemental Table S4. MMP, matrix metalloproteinase; TIMP, tissue inhibitor of metalloproteinase.

**
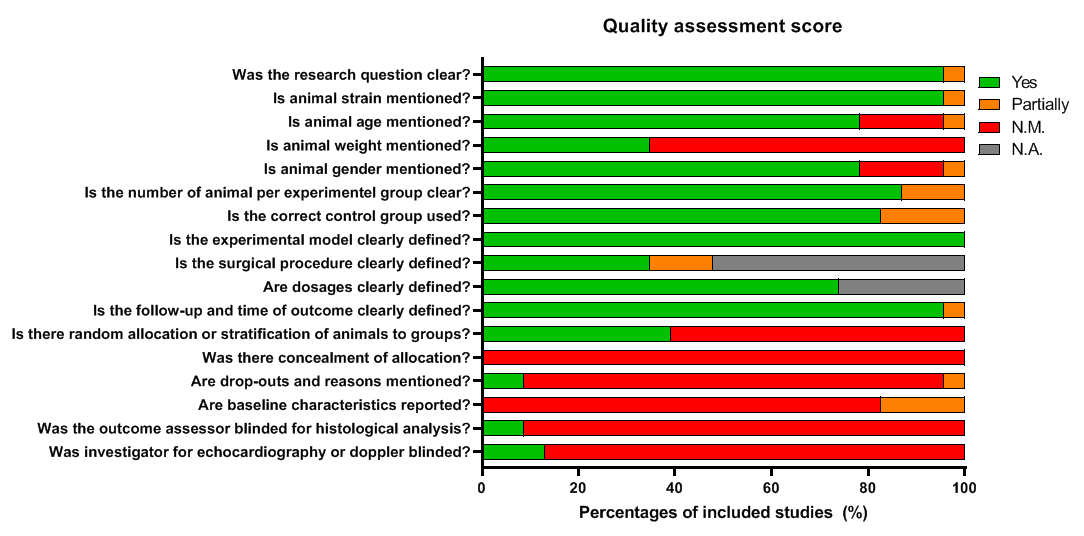
Supplemental Figure S3**. Quality assessment score. Shown is the quality assessment score (Risk of Bias tool) for the 23 included studies. This is an adapted version from Papazova et al., 2015.

**Supplemental Table S1-S4** can be found in individual excel files.

**Supplemental Table S1**. Study overview.

AngII, angiotensin 2; APN-KO, adiponectin knockout; DOCA, deoxycorticosterone acetate; HFD, high fat diet; SHR, spontaneous hypertensive rat; STZ, streptozotocin; TAC, transaortic constriction; UNX, uninephrectomy.

**Supplemental Table S2**. All extracted cardiac outcomes.

Diastolic cardiac outcomes of interest are highlighted in yellow; AngII, angiotensin 2; APN-KO, adiponectin knockout; cLVH, chronic left ventricular hypertrophy; dP/dt min, minimum rate of pressure change; DOCA, deoxycorticosterone acetate; E wave, peak early diastolic transmitral velocity; E/A, ratio between peak early diastolic transmitral velocity (E) and late (atrial) transmitral flow velocity (A); E/e’, ratio between peak early diastolic transmitral velocity (E) and early diastolic mitral annular velocity (e’); EDP, end diastolic pressure; EDPVR, end diastolic pressure volume relationship; HFD, high fat diet; IVRT, isovolumetric relaxation time; N, amount of animals; SD, standard deviation; SE, standard error; SEM, standard error of the mean; SHR, spontaneous hypertensive rat; STZ, streptozotocin; TAC, transaortic constriction; Tau, isovolumetric relaxation time constant; UNX, uninephrectomy; WT, wild type.

**Supplemental Table S3**. All extracted fibrotic outcomes.

AngII, angiotensin 2; AM, Azan Mallory; APN-KO, adiponectin knockout; cLVH, chronic left ventricular hypertrophy; DOCA, deoxycorticosterone acetate; HFD, high fat diet; MT, Masson’s Trichrome; N, amount of animals; SD, standard deviation; SE, standard error; SEM, standard error of the mean; SHR, spontaneous hypertensive rat; SR, Sirius Red; STZ, streptozotocin; TAC, transaortic constriction; UNX, uninephrectomy; VG, Van Gieson; WGA, Wheat Germ Agglutinin; WT, wild type.

**Supplemental Table S4**. All extracted MMP and TIMP outcomes.

AngII, angiotensin 2; APN-KO, adiponectin knockout; cLVH, chronic left ventricular hypertrophy; DOCA, deoxycorticosterone acetate; HFD, high fat diet; MMP, matrix metalloproteinase; N, amount of animals; SD, standard deviation; SE, standard error; SEM, standard error of the mean; SHR, spontaneous hypertensive rat; STZ, streptozotocin; TAC, transaortic constriction; TIMP, tissue inhibitor of metalloproteinase; UNX, uninephrectomy; WT, wild type.

**Supplemental Table S5**. Meta-analysis summary of all cardiac outcomes

|  |  | # Studies | N experimental | N control | **SMD** | 95% CI | P-value | I² |
| --- | --- | --- | --- | --- | --- | --- | --- | --- |
| **Tau** | **Hemodynamic** | 6 | 50 | 42 | **0.86** | 0.32, 1.41 | 0.002 | 30% |
|  | **Metabolic** | 4 | 42 | 38 | **1.02** | 0.19, 1.85 | 0.02 | 64% |
|  | **Total** | 10 | 82 | 80 | **0.94** | 0.49, 1.34 | <0.0001 | 43% |
| **IVRT** | **Hemodynamic** | 6 | 45 | 42 | **0.43** | -0.64, 1.49 | 0.43 | 80% |
|  | **Metabolic** | 6 | 45 | 35 | **1.16** | -0.18, 2.50 | 0.09 | 81% |
|  | **Total** | 12 | 90 | 77 | **0.77** | -0.05, 1.60 | 0.07 | 80% |
| **dP/dt min** | **Hemodynamic** | 3 | 22 | 17 | **-2.70** | -6.46, 1.05 | 0.16 | 88% |
|  | **Metabolic** | 4 | 40 | 38 | **-1.02** | -1.89, -0.14 | 0.02 | 67% |
|  | **Total** | 7 | 62 | 55 | **-1.18** | -2.17, -0.20 | 0.02 | 77% |
| **E wave** | **Hemodynamic** | 5 | 36 | 48 | **1.78** | -0.25, 3.82 | 0.09 | 88% |
|  | **Metabolic** | 4 | 16 | 18 | **0.40** | -0.74, 1.54 | 0.49 | 54% |
|  | **Total** | 10 | 52 | 66 | **0.86** | -0.29, 2.00 | 0.14 | 81% |
| **E/e'** | **Hemodynamic** | 9 | 106 | 104 | **0.89** | 0.32, 1.47 | 0.002 | 69% |
|  | **Metabolic** | 4 | 21 | 23 | **2.02** | 1.21, 2.83 | <0.00001 | 0% |
|  | **Total** | 13 | 127 | 127 | **1.18** | 0.64, 1.71 | <0.0001 | 68% |
| **E/A** | **Hemodynamic** | 8 | 56 | 52 | **1.14** | -0.27, 2.54 | 0.11 | 87% |
|  | **Metabolic** | 10 | 71 | 74 | **-0.30** | -1.41, 0.81 | 0.60 | 85% |
|  | **Total** | 18 | 127 | 126 | **0.28** | -0.57, 1.14 | 0.52 | 86% |
| **EDPVR** | **Hemodynamic** | 5 | 40 | 35 | **0.88** | 0.25, 1.50 | 0.006 | 32% |
|  | **Metabolic** | 2 | 17 | 15 | **0.95** | 0.20, 1.69 | 0.01 | 0% |
|  | **Total** | 7 | 57 | 50 | **0.85** | 0.43, 1.26 | <0.0001 | 1% |
| **EDP** | **Hemodynamic** | 9 | 64 | 56 | **0.40** | -0.06, 0.86 | 0.09 | 28% |
|  | **Metabolic** | 8 | 71 | 67 | **0.61** | -0.07, 1.29 | 0.08 | 70% |
|  | **Total** | 17 | 135 | 123 | **0.53** | 0.12, 0.94 | 0.01 | 55% |

CI, confidence interval; dP/dt min, minimum rate of pressure change; E wave, peak early diastolic transmitral velocity; E/A ratio between peak early diastolic transmitral velocity (E) and late (atrial) transmitral flow velocity (A); E/e’, ratio between peak early diastolic transmitral velocity (E) and early diastolic mitral annular velocity (e’); EDP, end diastolic pressure; EDPVR, end diastolic pressure volume relationship; HFD, high fat diet; I^2^, measurement of heterogeneity; IVRT, isovolumetric relaxation time; N/A, not available; SD, standard deviation; SE, standard error; SEM, standard error of the mean; SHR, spontaneous hypertensive rat; SMD, standardized mean difference; STZ, streptozotocin; TAC, transaortic constriction; Tau, isovolumetric relaxation time constant; UNX, uninephrectomy; WT, wild type.

**Supplemental Table S6**. Meta-analysis summary of all fibrotic outcomes

|  |  | # Studies | N experimental | N control | **SMD** | 95% CI | P-value | I² |
| --- | --- | --- | --- | --- | --- | --- | --- | --- |
| **Col1 mRNA** | **Hemodynamic** | 10 | 60 | 56 | **1.65** | 0.99, 2.31 | <0.00001 | 45% |
|  | **Metabolic** | 8 | 40 | 43 | **1.17** | 0.37, 1.97 | 0.004 | 56% |
|  | **Total** | 18 | 100 | 99 | **1.43** | 0.92, 1.94 | <0.00001 | 50% |
| **Col3 mRNA** | **Hemodynamic** | 10 | 60 | 56 | **1.75** | 0.68, 2.83 | 0.001 | 77% |
|  | **Metabolic** | 9 | 46 | 49 | **0.73** | 0.05, 1.42 | 0.04 | 54% |
|  | **Total** | 19 | 106 | 105 | **1.19** | 0.56, 1.82 | 0.0002 | 70% |
| **Col1 protein** | **Hemodynamic** | 5 | 31 | 31 | **1.13** | 0.21, 2.06 | 0.02 | 58% |
|  | **Metabolic** | 1 | 5 | 5 | **0.42** | -0.84, 1.68 | 0.52 | N/A |
|  | **Total** | 6 | 36 | 36 | **0.98** | 0.21, 1.74 | 0.01 | 50% |
| **Col3 protein** | **Hemodynamic** | 5 | 31 | 31 | **0.78** | -0.51, 2.07 | 0.23 | 78% |
|  | **Metabolic** | 1 | 5 | 5 | **0.51** | -0.76, 1.78 | 0.43 | N/A |
|  | **Total** | 6 | 36 | 36 | **0.69** | -0.35, 1.73 | 0.19 | 73% |
| **Collagen content** | **Hemodynamic** | 3 | 15 | 16 | **1.93** | -0.07, 3.92 | 0.06 | 75% |
|  | **Metabolic** | 4 | 18 | 19 | **0.65** | -0.44, 1.74 | 0.03 | 64% |
|  | **Total** | 7 | 33 | 35 | **1.10** | 0.13, 2.07 | 0.03 | 64% |
| **Fibrosis [IHC]** | **Hemodynamic** | 16 | 92 | 102 | **2.02** | 1.34, 2.71 | <0.00001 | 64% |
|  | **Metabolic** | 13 | 93 | 92 | **1.35** | 0.53, 2.18 | 0.001 | 80% |
|  | **Total** | 29 | 185 | 194 | **1.71** | 1.18, 2.24 | <0.00001 | 73% |

CI, confidence interval; Col1, Collagen type 1; Col3, Collagen type 3; I^2^, measurement of heterogeneity; IHC, immunohistochemistry; N, amount of animals; N/A, not available; SMD, standardized mean difference.

**Supplemental Table S7**. Meta-analysis summary of all MMP and TIMP outcomes

|  |  | # Studies | N experimental | N control | **SMD** | 95% CI | P-value | I² |
| --- | --- | --- | --- | --- | --- | --- | --- | --- |
| **MMP2 activity** | **Hemodynamic** | 10 | 57 | 69 | **0.68** | 0.14, 1.23 | 0.01 | 45% |
|  | **Metabolic** | 6 | 34 | 34 | **1.05** | -0.47, 2.56 | 0.18 | 82% |
|  | **Total** | 16 | 91 | 103 | **0.78** | 0.19, 1.37 | 0.010 | 67% |
| **MMP9 activity** | **Hemodynamic** | 5 | 26 | 25 | **0.75** | 0.16, 1.34 | 0.01 | 0% |
|  | **Metabolic** | 4 | 18 | 18 | **0.62** | -0.43, 1.68 | 0.25 | 49% |
|  | **Total** | 9 | 44 | 43 | **0.70** | 0.24, 1.16 | 0.003 | 0% |
| **MMP2 protein** | **Hemodynamic** | 2 | 10 | 10 | **2.35** | 0.57, 4.12 | 0.010 | 42% |
|  | **Metabolic** | 1 | 5 | 5 | **-1.78** | -3.37, -0.19 | 0.03 | N/A |
|  | **Total** | 3 | 15 | 15 | **1.03** | -1.90, 3.96 | 0.49 | 88% |
| **MMP9 protein** | **Hemodynamic** | 2 | 10 | 10 | **5.02** | 2.84, 7.21 | <0.00001 | 0% |
|  | **Metabolic** | 3 | 19 | 19 | **1.60** | 0.27, 2..93 | 0.02 | 64% |
|  | **Total** | 5 | 29 | 29 | **2.55** | 1.01, 4.10 | 0.001 | 72% |
| **TIMP1 protein** | **Hemodynamic** | 8 | 49 | 56 | **-0.05** | -0.45, 0.35 | 0.80 | 0% |
|  | **Metabolic** | 2 | 11 | 11 | **0.27** | -0.59, 1.12 | 0.54 | 0% |
|  | **Total** | 10 | 60 | 67 | **0.01** | -0.36, 0.37 | 0.98 | 0% |
| **TIMP2 protein** | **Hemodynamic** | 8 | 49 | 60 | **0.52** | 0.03, 1.01 | 0.04 | 30% |
|  | **Metabolic** | 2 | 11 | 11 | **-0.04** | -0.88, 0.80 | 0.93 | 0% |
|  | **Total** | 10 | 60 | 71 | **0.40** | -0.02, 0.83 | 0.06 | 24% |
| **MMP2 mRNA** | **Hemodynamic** | 9 | 60 | 55 | **1.33** | 0.13, 2.54 | 0.03 | 83% |
|  | **Metabolic** | 7 | 40 | 39 | **-0.56** | -1.60, 0.48 | 0.29 | 74% |
|  | **Total** | 16 | 100 | 94 | **0.48** | -0.40, 1.35 | 0.28 | 83% |
| **MMP8 mRNA** | **Hemodynamic** | 3 | 15 | 15 | **0.20** | -0.76, 1.17 | 0.68 | 37% |
|  | **Metabolic** | 5 | 24 | 25 | **0.35** | -1.35, 2.04 | 0.69 | 75% |
|  | **Total** | 8 | 39 | 40 | **0.29** | -0.64, 1.21 | 0.55 | 63% |
| **MMP9 mRNA** | **Hemodynamic** | 10 | 64 | 61 | **0.32** | -0.38, 1.03 | 0.37 | 68% |
|  | **Metabolic** | 9 | 52 | 51 | **0.19** | -0.73, 1.11 | 0.68 | 75% |
|  | **Total** | 19 | 116 | 112 | **0.27** | -0.28, 0.82 | 0.33 | 70% |
| **MMP11 mRNA** | **Hemodynamic** | 2 | 10 | 10 | **-0.52** | -1.45, 0.40 | 0.27 | 0% |
|  | **Metabolic** | 3 | 17 | 17 | **0.15** | -0.93, 1.22 | 0.79 | 52% |
|  | **Total** | 5 | 27 | 27 | **-0.11** | -0.85, 0.63 | 0.77 | 38% |
| **MMP12 mRNA** | **Hemodynamic** | 2 | 10 | 10 | **-0.02** | -1.53, 1.48 | 0.97 | 56% |
|  | **Metabolic** | 3 | 15 | 18 | **0.61** | -0.84, 2.06 | 0.41 | 70% |
|  | **Total** | 5 | 25 | 28 | **0.37** | -0.59, 1.33 | 0.45 | 59% |
| **MMP13 mRNA** | **Hemodynamic** | 2 | 9 | 11 | **0.99** | -0.86, 2.85 | 0.29 | 68% |
|  | **Metabolic** | N/A |  |  |  |  |  |  |
|  | **Total** | 2 | 9 | 11 | **0.99** | -0.86, 2.85 | 0.29 | 68% |
| **MMP14 mRNA** | **Hemodynamic** | 3 | 14 | 16 | **0.18** | -0.71, 1.07 | 0.69 | 26% |
|  | **Metabolic** | 3 | 16 | 17 | **-0.08** | -0.77, 0.61 | 0.82 | 0% |
|  | **Total** | 6 | 30 | 33 | **0.04** | -0.47, 0.55 | 0.89 | 0% |
| **MMP15 mRNA** | **Hemodynamic** | 2 | 10 | 10 | **-0.81** | -1.98, 0.36 | 0.17 | 31% |
|  | **Metabolic** | 3 | 16 | 17 | **-0.51** | -1.21, 0.19 | 0.16 | 0% |
|  | **Total** | 5 | 26 | 27 | **-0.62** | -1.19, -0.06 | 0.03 | 0% |
| **TIMP1 mRNA** | **Hemodynamic** | 8 | 51 | 46 | **1.18** | 0.43, 1.94 | 0.002 | 55% |
|  | **Metabolic** | 7 | 40 | 41 | **0.20** | -0.51, 0.91 | 0.58 | 52% |
|  | **Total** | 15 | 91 | 87 | **0.70** | 0.15, 1.25 | 0.01 | 60% |
| **TIMP2 mRNA** | **Hemodynamic** | 4 | 25 | 23 | **-0.09** | -1.22, 1.04 | 0.88 | 69% |
|  | **Metabolic** | 5 | 32 | 31 | **0.36** | -1.14, 0.41 | 0.36 | 53% |
|  | **Total** | 9 | 57 | 54 | **-0.25** | -0.87, 0.36 | 0.42 | 57% |
| **TIMP3 mRNA** | **Hemodynamic** | 2 | 12 | 11 | **-0.32** | -1.15, 0.50 | 0.44 | 0% |
|  | **Metabolic** | 4 | 21 | 21 | **-0.29** | -2.50, 1.92 | 0.80 | 87% |
|  | **Total** | 6 | 33 | 32 | **-0.28** | -1.54, 0.98 | 0.67 | 78% |
| **TIMP4 mRNA** | **Hemodynamic** | 5 | 25 | 24 | **0.08** | -0.60, 0.76 | 0.82 | 21% |
|  | **Metabolic** | 3 | 16 | 17 | **-1.23** | -2.01, -0.44 | 0.002 | 0% |
|  | **Total** | 8 | 41 | 41 | **-0.42** | -1.06, 0.22 | 0.20 | 43% |

CI, confidence interval; I^2^, measurement of heterogeneity; IHC, immunohistochemistry; MMP, matrix metalloproteinase; N, amount of animals; N/A, not available; SMD, standardized mean difference; TIMP, tissue inhibitor of metalloproteinase.

**Supplemental Table S8**. MEDLINE and Embase search syntax

.

| **Search** | **MEDLINE search syntax** | **Hits** |
| --- | --- | --- |
| #1 | ("Heart Failure"[Mesh] OR "Heart Failure, Diastolic"[Mesh]) NOT "Heart Failure, Systolic"[Mesh] | 117567 |
| #2 | "Ventricular function"[Mesh] OR "Ventricular dysfunction"[Mesh] | 91787 |
| #3 | "Endomyocardial Fibrosis"[Mesh] | 1978 |
| #4 | "Myocardial Contraction"[Mesh] | 76686 |
| #5 | "Heart ventricles"[Mesh] | 80460 |
| #6 | "Heart failure with preserved ejection fraction"[tiab] OR "HFpEF"[tiab] OR "Left ventricle diastolic dysfunction"[tiab] OR "Diastolic Function"[tiab] OR "LVDD"[tiab] OR "Heart failure with normal ejection fraction"[tiab] OR "HFNEF"[tiab] OR "Diastolic Heart Failure"[tiab] | 15403 |
| #7 | (Heart[tiab] OR Cardiac[tiab] OR ventric*[tiab] OR myocardia*[tiab]) AND remode*[tiab] | 32204 |
| #8 | (Heart[tiab] OR Cardiac[tiab] OR ventric*[tiab] OR myocardia*[tiab]) AND function*[tiab] | 282795 |
| #9 | (Heart[tiab] OR Cardiac[tiab] OR ventric*[tiab] OR myocardia*[tiab]) AND fibrosis*[tiab] | 24132 |
| #10 | #1 OR #2 OR #3 OR #4 OR #5 OR #6 OR #7 OR #8 OR #9 | 516296 |
| #11 | (Heart Function Tests[Mesh] OR Hemodynamic monitoring[Mesh]) | 546505 |
| #12 | Angiograp*[tiab] OR cardiac output*[tiab] OR Ejection Fraction[tiab] OR Echocardiograp*[tiab] OR Echo[tiab] OR Left ventric* end-diastolic volume index[tiab] OR LVEDVI[tiab] | 1378 |
| #13 | #11 OR #12 | 546954 |
| #14 | #10 AND #13 | 150860 |
| #15 | "Fibrosis"[Mesh] OR "Extracellular Matrix"[Mesh] OR "Extracellular Matrix Proteins"[Mesh] OR "Basement Membrane"[Mesh] | 375536 |
| #16 | "Matrix Metalloproteinases"[Mesh] OR "Tissue Inhibitor of Metalloproteinases"[Mesh] OR "Collagen"[Mesh] OR "ADAMTS Proteins"[Mesh] OR "ADAM Proteins"[Mesh] | 169979 |
| #17 | Fibrot*[tiab] Fibros*[tiab] OR Connective tissue[tiab] OR Extracellular Matrix[tiab] OR Extracellular Matrix Proteins[tiab] OR Basement Membrane*[tiab] OR ECM[tiab] | 192708 |
| #18 | Matrix Metalloproteinase*[tiab] OR "Tissue Inhibitor* of Metalloproteinase*"[tiab] OR Collagen*[tiab] OR ADAMTS Protein*[tiab] OR ADAM Protein*[tiab] OR MMP*[tiab] OR TIMP*[tiab] OR ADAM[tiab] OR ADAMTS[tiab] | 285163 |
| #19 | #15 OR #16 OR #17 OR #18 | 648286 |
| #20 | #14 AND #19 | 3983 |

| **Search** | **Embase search syntax** | **Hits** |
| --- | --- | --- |
| #1 | 'heart failure'/exp OR 'diastolic heart failure'/exp NOT 'systolic heart failure'/exp | 498684 |
| #2 | 'heart ventricle function'/exp OR 'heart ventricle dysfunction'/exp | 18003 |
| #3 | 'heart muscle fibrosis'/exp | 22328 |
| #4 | 'heart contraction'/exp | 65438 |
| #5 | 'heart ventricle'/exp | 141883 |
| #6 | Heart failure with preserved ejection fraction:ti,ab OR HFpEF:ti,ab OR Left ventricle diastolic dysfunction:ti,ab OR Diastolic Function:ti,ab OR LVDD:ti,ab OR Heart failure with normal ejection fraction:ti,ab OR HFNEF:ti,ab OR Diastolic Heart Failure:ti,ab | 40691 |
| #7 | (Heart:ti,ab OR Cardiac:ti,ab OR ventric*:ti,ab OR myocardia*:ti,ab) AND remode*:ti,ab | 55140 |
| #8 | (Heart:ti,ab OR Cardiac:ti,ab OR ventric*:ti,ab OR myocardia*:ti,ab) AND function*:ti,ab | 432410 |
| #9 | (Heart:ti,ab OR Cardiac:ti,ab OR ventric*:ti,ab OR myocardia*:ti,ab) AND fibrosis:ti,ab | 41343 |
| #10 | #1 OR #2 OR #3 OR #4 OR #5 OR #6 OR #7 OR #8 OR #9 | 976303 |
| #11 | 'heart function test'/exp OR 'hemodynamic monitoring'/exp | 162847 |
| #12 | Angiograp*:ti,ab OR cardiac output*:ti,ab OR Ejection Fraction:ti,ab OR Echocardiograp*:ti,ab OR Echo:ti,ab OR Left ventric* end-diastolic volume index:ti,ab OR LVEDVI:ti,ab | 6686 |
| #13 | #11 OR #12 | 168984 |
| #14 | #10 AND #13 | 42715 |
| #15 | 'Fibrosis'/exp OR 'extracellular matrix'/exp OR 'extracellular matrix protein'/exp OR 'basement membrane'/exp | 396593 |
| #16 | 'Matrix metalloproteinase'/exp OR 'tissue inhibitor of metalloproteinase'/exp OR 'collagen'/exp OR 'ADAM protein'/exp | 250467 |
| #17 | Fibro*:ti,ab OR Connective tissue:ti,ab OR Extracellular Matrix:ti,ab OR Extracellular Matrix Proteins:ti,ab OR Basement Membrane*:ti,ab OR ECM:ti,ab | 84309 |
| #18 | matrix metalloproteinase*:ti,ab OR Tissue Inhibitor* of Metalloproteinase*:ti,ab OR Collagen*:ti,ab OR ADAMTS Protein*:ti,ab OR ADAM Protein*:ti,ab OR MMP*:ti,ab OR TIMP*:ti,ab OR ADAMTS:ti,ab OR ADAM:ti,ab | 198387 |
| #19 | #15 OR #16 OR #17 OR #18 | 714610 |
| #20 | #14 AND #19 | 1641 |

Supplemental Table S9. PRISMA checklist

| **Section/topic** | **#** | **Checklist item** | **Reported on page #** |
| --- | --- | --- | --- |
| **TITLE** | | |  |
| Title | 1 | Identify the report as a systematic review, meta-analysis, or both. | 1 |
| **ABSTRACT** | | |  |
| Structured summary | 2 | Provide a structured summary including, as applicable: background; objectives; data sources; study eligibility criteria, participants, and interventions; study appraisal and synthesis methods; results; limitations; conclusions and implications of key findings; systematic review registration number. | 1 |
| **INTRODUCTION** | | |  |
| Rationale | 3 | Describe the rationale for the review in the context of what is already known. | 2 |
| Objectives | 4 | Provide an explicit statement of questions being addressed with reference to participants, interventions, comparisons, outcomes, and study design (PICOS). | 2 |
| **METHODS** | | |  |
| Protocol and registration | 5 | Indicate if a review protocol exists, if and where it can be accessed (e.g., Web address), and, if available, provide registration information including registration number. | 12 |
| Eligibility criteria | 6 | Specify study characteristics (e.g., PICOS, length of follow-up) and report characteristics (e.g., years considered, language, publication status) used as criteria for eligibility, giving rationale. | 12-13 |
| Information sources | 7 | Describe all information sources (e.g., databases with dates of coverage, contact with study authors to identify additional studies) in the search and date last searched. | 12-13 |
| Search | 8 | Present full electronic search strategy for at least one database, including any limits used, such that it could be repeated. | 12-13 and Supp table S8 |
| Study selection | 9 | State the process for selecting studies (i.e., screening, eligibility, included in systematic review, and, if applicable, included in the meta-analysis). | 12-13 |
| Data collection process | 10 | Describe method of data extraction from reports (e.g., piloted forms, independently, in duplicate) and any processes for obtaining and confirming data from investigators. | 14 |
| Data items | 11 | List and define all variables for which data were sought (e.g., PICOS, funding sources) and any assumptions and simplifications made. | 13-14 |
| Risk of bias in individual studies | 12 | Describe methods used for assessing risk of bias of individual studies (including specification of whether this was done at the study or outcome level), and how this information is to be used in any data synthesis. | 13-14 and Supp Fig S3 |
| Summary measures | 13 | State the principal summary measures (e.g., risk ratio, difference in means). | 14 |
| Synthesis of results | 14 | Describe the methods of handling data and combining results of studies, if done, including measures of consistency (e.g., I^2^) for each meta-analysis. | 14 |

| **Section/topic** | **#** | **Checklist item** | **Reported on page #** |
| --- | --- | --- | --- |
| Risk of bias across studies | 15 | Specify any assessment of risk of bias that may affect the cumulative evidence (e.g., publication bias, selective reporting within studies). | 4, 13-14 |
| Additional analyses | 16 | Describe methods of additional analyses (e.g., sensitivity or subgroup analyses, meta-regression), if done, indicating which were pre-specified. | 4-10 |
| **RESULTS** | | |  |
| Study selection | 17 | Give numbers of studies screened, assessed for eligibility, and included in the review, with reasons for exclusions at each stage, ideally with a flow diagram. | 2-3 and Figure 1 |
| Study characteristics | 18 | For each study, present characteristics for which data were extracted (e.g., study size, PICOS, follow-up period) and provide the citations. | 2-4 |
| Risk of bias within studies | 19 | Present data on risk of bias of each study and, if available, any outcome level assessment (see item 12). | 3-4 and Supp Fig S3 |
| Results of individual studies | 20 | For all outcomes considered (benefits or harms), present, for each study: (a) simple summary data for each intervention group (b) effect estimates and confidence intervals, ideally with a forest plot. | 2-10 |
| Synthesis of results | 21 | Present results of each meta-analysis done, including confidence intervals and measures of consistency. | 2-10 and Supp Table S5 |
| Risk of bias across studies | 22 | Present results of any assessment of risk of bias across studies (see Item 15). | 3-4 and 12-13 |
| Additional analysis | 23 | Give results of additional analyses, if done (e.g., sensitivity or subgroup analyses, meta-regression [see Item 16]). | 2-10 |
| **DISCUSSION** | | |  |
| Summary of evidence | 24 | Summarize the main findings including the strength of evidence for each main outcome; consider their relevance to key groups (e.g., healthcare providers, users, and policy makers). | 10 |
| Limitations | 25 | Discuss limitations at study and outcome level (e.g., risk of bias), and at review-level (e.g., incomplete retrieval of identified research, reporting bias). | 10-13 |
| Conclusions | 26 | Provide a general interpretation of the results in the context of other evidence, and implications for future research. | 14-15 |
| **FUNDING** | | |  |
| Funding | 27 | Describe sources of funding for the systematic review and other support (e.g., supply of data); role of funders for the systematic review. | 15 |
